# Linking higher amyloid beta 1‐38 (Aβ(1‐38)) levels to reduced Alzheimer's disease progression risk

**DOI:** 10.1002/alz.14545

**Published:** 2025-01-27

**Authors:** Luisa Sophie Schneider, Silka Dawn Freiesleben, Gerard van Breukelen, Xiao Wang, Frederic Brosseron, Michael T. Heneka, Stefan Teipel, Luca Kleineidam, Melina Stark, Nina Roy‐Kluth, Michael Wagner, Annika Spottke, Matthias Schmid, Sandra Roeske, Christoph Laske, Matthias H. Munk, Robert Perneczky, Boris‐Stephan Rauchmann, Katharina Buerger, Daniel Janowitz, Emrah Düzel, Wenzel Glanz, Frank Jessen, Ayda Rostamzadeh, Jens Wiltfang, Claudia Bartels, Ingo Kilimann, Anja Schneider, Klaus Fliessbach, Josef Priller, Eike Jakob Spruth, Julian Hellmann‐Regen, Oliver Peters

**Affiliations:** ^1^ Department of Psychiatry and Neuroscience Charité – Universitätsmedizin Berlin corporate member of Freie Universität Berlin Humboldt‐Universität zu Berlin Berlin Germany; ^2^ Memory Clinic and Dementia Prevention Center Experimental and Clinical Research Center (ECRC) Berlin Germany; ^3^ German Center for Neurodegenerative Diseases (DZNE) Berlin Germany; ^4^ Department of Methodology & Statistic Maastricht University Faculty of Psychology and Neuroscience and CAPHRI Care and Public Health Research Institute Maastricht Netherlands; ^5^ German Center for Neurodegenerative Diseases (DZNE) Bonn Germany; ^6^ Luxembourg Centre for Systems Biomedicine (LCSB) University of Luxembourg Belvaux Luxembourg; ^7^ German Center for Neurodegenerative Diseases (DZNE) Rostock Germany; ^8^ Department of Psychosomatic Medicine Rostock University Medical Center Rostock Germany; ^9^ Department for Cognitive Disorders and Old Age Psychiatry University Hospital Bonn Bonn Germany; ^10^ Institute for Medical Biometry University Hospital Bonn Bonn Germany; ^11^ Department of Neurology University of Bonn Bonn Germany; ^12^ Section for Dementia Research Hertie Institute for Clinical Brain Research and Department of Psychiatry and Psychotherapy University of Tübingen Tübingen Germany; ^13^ German Center for Neurodegenerative Diseases (DZNE) Tübingen Germany; ^14^ Department of Psychiatry and Psychotherapy University of Tübingen Tübingen Germany; ^15^ German Center for Neurodegenerative Diseases (DZNE) Munich Germany; ^16^ Department of Psychiatry and Psychotherapy University Hospital LMU Munich Munich Germany; ^17^ Ageing Epidemiology Research Unit (AGE) School of Public Health Imperial College London South Kensington Campus London UK; ^18^ Munich Cluster for Systems Neurology (SyNergy) Munich Munich Germany; ^19^ Sheffield Institute for Translational Neuroscience (SITraN) University of Sheffield Sheffield UK; ^20^ Department of Neuroradiology University Hospital LMU Munich Germany; ^21^ Institute for Stroke and Dementia Research (ISD) University Hospital LMU Munich Munich Germany; ^22^ German Center for Neurodegenerative Diseases (DZNE) Magdeburg Germany; ^23^ Institute of Cognitive Neurology and Dementia Research (IKND) Otto‐von‐Guericke University Magdeburg Germany; ^24^ Department of Psychiatry University of Cologne Medical Faculty Cologne Germany; ^25^ Excellence Cluster on Cellular Stress Responses in Aging‐Associated Diseases (CECAD) University of Cologne Köln Germany; ^26^ German Center for Neurodegenerative Diseases (DZNE) Goettingen Germany; ^27^ Department of Medical Sciences Neurosciences and Signaling Group Institute of Biomedicine (iBiMED) University of Aveiro Aveiro Portugal; ^28^ Department of Psychiatry and Psychotherapy University Medical Center Goettingen University of Goettingen Goettingen Germany; ^29^ Centre for Clinical Brain Sciences University of Edinburgh and UK DRI Edinburgh UK; ^30^ Department of Psychiatry and Psychotherapy School of Medicine Technical University of Munich Munich Germany

**Keywords:** AD conversion risk, Alzheimer's disease, Aβ(1‐38), cerebrospinal fluid, cognitive decline, neurotoxicity, protective factor, shorter Aβ peptides

## Abstract

**INTRODUCTION:**

The beneficial effects of amyloid beta 1‐38, or Aβ(1‐38), on Alzheimer's disease (AD) progression in humans in vivo remain controversial. We investigated AD patients' cerebrospinal fluid (CSF) Aβ(1‐38) and AD progression.

**METHODS:**

Cognitive function and diagnostic change were assessed annually for 3 years in 177 Aβ‐positive participants with subjective cognitive decline (SCD), mild cognitive impairment (MCI), and dementia from the German Center for Neurodegenerative Diseases (DZNE) longitudinal cognitive impairment and dementia study (DELCODE) cohort using the Mini‐Mental State Examination (MMSE), Preclinical Alzheimer's Cognitive Composite (PACC), Clinical Dementia Rating (CDR), and National Institute of Neurological and Communicative Disorders and Stroke–Alzheimer's Disease and Related Disorders Association (NINCDS‐ADRDA) criteria. Mixed linear and Cox regression analyses were conducted. CSF was collected at baseline.

**RESULTS:**

Higher Aβ(1‐38) levels were associated with slower PACC (*p *= 0.001) and slower CDR Sum of Boxes (CDR‐SB) (*p *= 0.002) but not MMSE decline. Including Aβ(1‐40) beyond Aβ(1‐38) in the model confirmed an association of Aβ(1‐38) with slower PACC decline (*p *= 0.005), but not with CDR‐SB or MMSE decline. In addition, higher Aβ(1‐38) baseline levels were associated with a reduced dementia conversion risk.

**DISCUSSION:**

Further research is needed to understand the role of Aβ(1‐38) in AD and its potential for future therapeutic strategies.

**Highlights:**

- This study not only replicates but also extends the existing findings on the role of Aβ(1‐38) (amyloid beta 1‐38) in Alzheimer's disease (AD) in humans in vivo.
- Higher baseline Aβ(1‐38) levels were associated with a decreased risk of conversion to AD dementia in subjective cognitive decline (SCD) and mild cognitive impairment (MCI).
- Different linear‐mixed regression models suggest an association between higher Aβ(1‐38) baseline levels and slower Preclinical Alzheimer's Cognitive Composite (PACC) and Clinical Dementia Rating Sum of Boxes (CDR‐SB) decline.
- Including Aβ(1‐40) beyond Aβ(1‐38) in the model confirmed a link between Aβ(1‐38) and PACC decline, but showed no association of Aβ(1‐38) on CDR‐SB and Mini‐Mental State Examination (MMSE) decline.
- The impact of short Aβ isoforms in AD progression might have been under‐investigated
- These findings underscore the urgent need for additional research on the role of these shorter Aβ peptides in AD, as they may hold key insights for future therapeutic strategies.

## 1 BACKGROUND

Alzheimer's disease (AD) is the leading cause of dementia, representing the largest share of the 57.4 million people living with dementia worldwide.^1^ One key mechanism involved in the pathophysiology of AD is the increased aggregation of long amyloid beta (Aβ) peptides, such as Aβ(1‐42) in the cerebrospinal fluid (CSF) into neurotoxic cerebral Aβ plaques.^2^, ^3^ Furthermore, Aβ is deemed responsible for initiating neurotoxic and pathologic downstream effects, including phosphorylation at threonine‐181 of tau (p‐tau181), resulting in neuronal decay.^4^, ^5^ However, Aβ peptides exist in varying lengths and quantities, yet their specific effects on AD neuropathology remain elusive.^6^, ^7^ In particular, biomarker research on the role of short Aβ peptides in AD is sparse. Recent findings suggest that short Aβ peptides such as Aβ(1‐38) in the CSF may lower the risk of AD progression.^8^, ^9^, ^10^

It is estimated that ≈90% of cerebral Aβ is composed of Aβ(1‐40), whereas Aβ(1‐37), Aβ(1‐38), Aβ(1‐39), and Aβ(1‐42) account for 5%–20% of the total Aβ pool.^6^ Preclinical in vivo and in vitro studies have demonstrated that the production of shorter Aβ peptides shifts toward producing longer, neurotoxic Aβ peptides, such as Aβ(1‐42) in AD.^4^, ^11^ Moreover, several preclinical studies suggest that short Aβ peptides, including Aβ(1‐38), are significantly less toxic compared to longer forms such as Aβ(1‐42),^10^, ^11^, ^12^ and that raising short Aβ peptide levels, such as Aβ(1‐38) levels, can reduce Aβ deposition in mouse models in vivo.^10^ In addition, a series of recent in vitro and in vivo biophysical experiments in mice, *Caenorhabditis elegans* worms, and human connective tissue have shown that Aβ(1‐38) interferes with Aβ(1‐42) aggregation and reverses impaired long‐term potentiation mediated by Aβ(1‐42).^9^

By contrast, data on the potential protective effects of Aβ(1‐38) in humans in vivo are sparse, although promising data are starting to emerge. For example, Cullen et al. have recently reported using the Swedish BioFINDER and the North American Alzheimer's Disease Neuroimaging Initiative (ADNI) cohorts that higher CSF Aβ(1‐38) levels correlate with a lower risk of conversion to AD dementia, and a slower cognitive decline when adjusted for demographics, Aβ(1‐42), and p‐tau181 in individuals with subjective cognitive decline (SCD), mild cognitive impairment (MCI) due to AD, and AD dementia.^8^ Thus, replicating and extending these findings is highly warranted, considering the promising results and the paucity of data on the potential protective effects of Aβ(1‐38) in humans in vivo.

The purpose of the present study was first to replicate and second to extend the current knowledge on the role of Aβ(1‐38) in AD in humans in vivo. First, given that several studies have shown that baseline cognitive status affects the rate of cognitive and functional decline,^13^, ^14^, ^15^ the present study aims to add control for participants' screening diagnosis to investigate the role of Aβ(1‐38) on AD‐related decline. Second, the previous study focused on Aβ(1‐38) adjusted for Aβ(1‐42) and p‐tau181. Our study also aims to adjust for Aβ(1‐40) to explore the potential dynamic effects of the full range of AD‐relevant and available Aβ peptides. Third, unlike in the previous study, where separate mixed linear regression (MLR) models were fitted to examine the impact of Aβ(1‐38) adjusted for Aβ(1‐42) and p‐tau181 on cognitive change, our approach entails examining all biomarker combinations of Aβ(1‐38), Aβ(1‐40), Aβ(1‐42), and p‐tau181 in one model and thereby statistically determine which biomarker combination best fits the data. In this vein, we aim to statistically determine which biomarker combination of Aβ(1‐42), Aβ(1‐40), and p‐tau181 with Aβ(1‐38) best relates to cognitive change using a unique statistical approach, which is discussed extensively elsewhere.^16^ Specifically, focusing on Aβ(1‐38), we investigated which combination of CSF Aβ(1‐38), Aβ(1‐42), p‐tau181, and Aβ(1‐40) biomarkers best describes the cognitive change and risk of conversion to AD dementia in individuals along the AD spectrum.

## 2 METHODS

### 2.1 Participants

Participants ≥60 years of age were enrolled from the German DZNE multicenter Longitudinal Cognitive Impairment and Dementia (DELCODE) observational study.^17^ Participants were recruited in 10 memory clinics throughout Germany and included SCD, MCI, and AD dementia patients, as well as control participants comprising healthy controls (HCs) and first‐degree relatives of patients with AD dementia. For the present study, only SCD, MCI, and AD dementia patients were included. Groups were defined by established research criteria.^17^, ^18^, ^19^ In short, the SCD group included participants with self‐experienced cognitive decline without objective cognitive impairment (i.e., >1.5 standard deviation [SD] below the age‐ and sex‐matched mean in all subtests of the Consortium to Establish a Registry for Alzheimer's Disease [CERAD] neuropsychological test battery). The MCI group included amnestic MCI participants who obtained <−1.5 SD below the age‐, education years‐, and sex‐adjusted mean in the delayed word‐list recall trial of the CERAD, and who did not meet the criteria for AD dementia according to the National Institute of Neurological and Communicative Disorders and Stroke (NINCDS) and the Alzheimer's Disease and Related Disorders Association (ADRDA).^18^ Allocation to the AD dementia group was defined as having a Mini‐Mental State Examination (MMSE) score of ≥18 points and fulfilling the revised mild AD dementia criteria according to NINCDS‐ADRDA.^19^ For the present study, participants were selected based on the A/T/N research classification system for AD diagnosis established by the National Institute on Aging and Alzheimer's Association (NIA‐AA).^20^, ^21^ Cullen and colleagues^8^ used the Aβ(1‐42)/p‐tau181 ratio to identify A+ individuals. To ensure comparability with their study, the current work also applied the Aβ(1‐42)/p‐tau181 ratio to identify A+ individuals. Accordingly, only participants with a positive Aβ42/p‐tau181 ratio (A+) of >9.68 at baseline were included, resulting in *N* = 177, with SCD = 56, MCI = 60, and AD = 61.

**RESEARCH IN CONTEXT**

1. **Systematic review**: The literature was reviewed using conventional sources (e.g., PubMed, Google Scholar). The research consensus is that long Aβ(1‐42) (amyloid beta 1‐42) peptides are neurotoxic pathological hallmarks of Alzheimer's disease (AD). Of interest, short Aβ peptides such as Aβ(1‐38) appear to be less neurotoxic and may even counteract AD neuropathology. However, their role in AD in humans in vivo has been largely overlooked.
2. **Interpretation**: Higher baseline levels of Aβ(1‐38) were linked to reduced risk of developing Alzheimer's dementia in subjective cognitive decline (SCD) and mild cognitive impairment (MCI). Various mixed‐linear regression models suggested an association between higher Aβ(1‐38) baseline levels and slower Preclinical Alzheimer's Cognitive Composite (PACC) and decline in Clinical Dementia Rating Sum of Boxes (CDR‐SB). Including Aβ(1‐40) beyond Aβ(1‐38) in the model confirmed a link between Aβ(1‐38) and PACC performance but showed no association of Aβ(1‐38) on CDR‐SB and Mini‐Mental State Examination (MMSE) decline. Our results show that the role of Aβ(1‐38) in AD pathophysiology warrants further investigation.
3. **Future directions**: These findings emphasize the urgent need for additional research on the role of these shorter Aβ peptides in AD, as they may offer critical insights for future therapeutic strategies.

### 2.2 Biomarkers

CSF was collected at baseline, and the aliquoted samples were stored at −80°C. The assessment of CSF AD biomarkers, that is, Aβ(1‐38), Aβ(1‐40), Aβ(1‐42), and p‐tau181, was conducted in the DZNE core research facility in Bonn using standard commercial kits, including the V‐PLEX Aβ Peptide Panel 1 (6E10) Kit (K15200E) and the Innotest Phospho‐Tau(1818P) (81581; Fujirebio Germany GmbH, Hannover, Germany). Biomarkers were measured in picograms per milliliter (pg/mL), with cutoff values determined based on a sample of 527 baseline DELCODE participants who had available CSF data. The cutoff values were as follows: Aβ(1‐42) ≤638.7, Aβ(1‐42)/(1‐40) ≤0.08, Aβ(1‐42)/p‐tau181 >9.68, and p‐tau181 >73.65. Detailed information on establishing cutoff values in DELCODE is available elsewhere.^17^

### 2.3 Clinical measures

Cognitive function was assessed annually with the MMSE (score range 0–30; higher scores indicate better performance) and the CERAD test battery (multi‐domain cognitive assessment; raw scores converted to *z*‐scores). Based on these neuropsychological tests, a Preclinical Alzheimer's Cognitive Composite (PACC) score was computed by averaging the *z*‐scores of the MMSE, the Wechsler Memory Scale–Revised (WMS‐R) Logical Memory Story Delayed Recall test (score 0–25), the Symbol‐Digit‐Modalities‐Test (score 0–90), the sum of the Free and Total Recall trials (score 0–96) of the Free Cued and Selective Recall Test (FCRST), and the Categorical Fluency Animals and Food test (sum of correct words).^17^, ^22^, ^23^ Conversion to AD dementia was determined based on the Clinical Dementia Rating (CDR) scale score of ≥1 and NINCDS‐ADRDA criteria, resulting in a binary outcome for conversion (i.e., 1 = yes; 0 = no). Similarly, clinical decline was assessed using the CDR Sum of Boxes (CDR‐SB; range 0–18). Higher scores on the CDR‐SB indicate greater impairment.

### 2.4 Statistical analyses

Statistical analyses were performed using SPSS.^24^ Graphs were created with GraphPad software.^25^ All biomarkers were naturally log‐transformed to reduce skewness. All statistical analysis models were adjusted for age, sex, and years of education (hereinafter referred to as education). In view of very large percentages of missingness for one or more outcomes from follow‐up 4, all analyses were carried out until and including follow‐up 3.

#### 2.4.1 Aβ(1‐38) and longitudinal cognitive functioning

**FIGURE 1:**
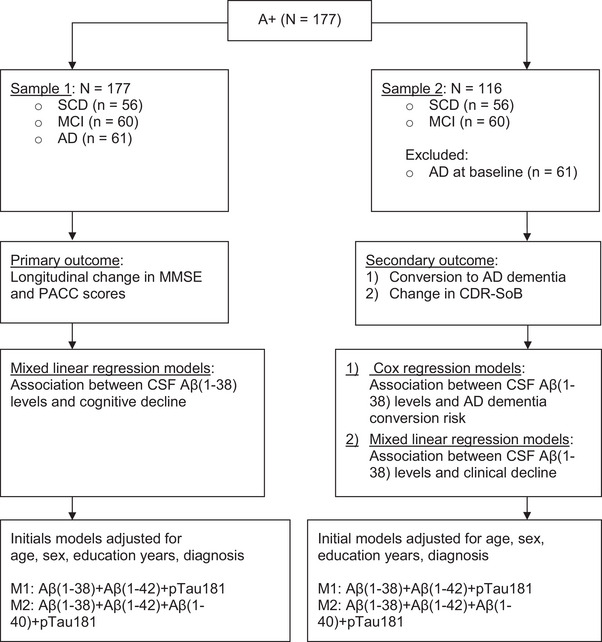
Flowchart: Statistical analyses in sample 1 (i.e., MMSE and PACC) and sample 2 (i.e., conversion to dementia, CDR‐SB). From *N* = 177, two samples were created to conduct statistical analyses on cognitive change and risk of conversion to AD dementia within 3 years. Sample 1 included individuals with SCD, MCI, or AD, whereas sample 2 focused on MCI and SCD. MLR examined the link between Aβ(1‐38) and cognitive decline using the PACC and MMSE scores. Cox regression assessed conversion risk to AD dementia, with MLR applied to clinical decline in the CDR‐SB. Two adjustment models were used: model 1 (M1) excluded Aβ(1‐40), whereas model 2 (M2) included it. A+, positive Aβ(1‐42)/p‐tau181 ratio; AD, Alzheimer's disease dementia; Aβ(1‐38), amyloid beta 1‐38; CDR‐SB, Clinical Dementia Rating Sum of Boxes; M1, model 1; M2, model 2; MCI, mild cognitive impairment; MLR, mixed linear regression; MMSE, Mini‐Mental State Examination; PACC, Preclinical Alzheimer's Cognitive Composite; p‐tau181, tau protein phosphorylated at threonine 181; SCD, subjective cognitive decline.

To examine the association between Aβ(1‐38) baseline levels and cognitive function change over time, indicated by MMSE and PACC score change, adjusted for Aβ(1‐42), p‐tau181, sex, education, baseline age, screening diagnosis (SCD, MCI, AD), and additionally also for Aβ(1‐40), MLR modeling was performed on the total sample, referred to as sample 1, of *N* = 177 patients for the MMSE and *N* = 153 for PACC (Figure 1). For all predictor variables, we tested their interaction with time which, if present, would imply a relation between the predictor and cognitive change from baseline.

##### Modeling procedure for the repeated measures of the MMSE, PACC, and CDR‐SB

MMSE, PACC and CDR‐SB score changes were analyzed with MLR. To replicate previous results that investigated Aβ(1‐38) and Aβ(1‐40) in separate models,^8^ Aβ(1‐40) was initially omitted (model 1 hereafter referred to as M1), but subsequently added as predictor (model 2 hereafter referred to as M2); see Figure 1. The fixed (predictor) part of the models consisted of age, sex, education (in years), diagnosis group (using dummy coding with AD as reference category), the biomarkers [i.e., Aβ(1‐42), Aβ(1‐40), p‐tau181, Aβ(1‐38)], time in visit numbers (using dummy coding with baseline visit as reference category) or time in days since baseline (as a continuous variable), and the interaction of all predictors with time. As said before, biomarkers were log‐transformed. A random intercept was included to account for possible outcome differences between the participating test centers; that is, the study sites. The random within‐center (covariance structure) part of the mixed model was unstructured; that is, the residual outcome variance was allowed to differ between time points, and the residual correlation was allowed to vary between pairs of time points. Using restricted maximum likelihood (REML) estimation, model simplifications were looked for.^16^ Specifically, the reduction of the fixed parts was tested by deleting nonsignificant (n.s.) interactions with time. Furthermore, M1 and M2 were run twice, once with days since baseline as a continuous time variable (thus assuming a linear time effect) and once with visit number as a categorical time variable using dummy coding (thus allowing for a nonlinear time effect). In both cases, REML was used, and the model was reduced by dropping n.s. interactions with time stepwise. Given that the predicted MMSE trajectory per diagnostic group showed a monotonically decreasing, roughly linear pattern per follow‐up and that there was a strong between‐person variation in time gaps between visits (i.e., between 9 and 29 months), linear models entailing days since baseline as a time variable were considered primary and reported in the following. However, these two approaches to time modeling sometimes resulted in different final models—that is, time‐as‐factor models resulting in final models with different predictors and interactions compared to time‐as‐continuous variable models. To address this, we performed Likelihood‐Ratio (LR) testing of the initial and final linear against nonlinear models, using Maximum Likelihood (ML) estimation. This process allowed us to statistically determine whether time‐as‐factor models or time‐as‐continuous‐variable models provided a better description of the data. When the final models differed in their fixed part, that is, including different predictors and interactions, we focused on comparing the initial models.

#### 2.4.2 Aβ(1‐38) and dementia conversion risk and clinical disease progression

##### Cox regression

Cox regression analyses were conducted in sample 2 (see Figure 1, right‐hand side) to assess the relation between Aβ(1‐38) levels at baseline and the risk of conversion to AD dementia. For this analysis, AD dementia participants were excluded as they had already converted to dementia before the study entry. Time was calculated based on the study entry date and event date, treating conversions as events and dropouts as censorings. The proportional hazards (PHs) assumption was tested by including a time‐dependent covariate, specifically an interaction of time with a predictor showing a significant effect in the analysis without interaction. Possible outcome differences between the testing sites were accounted for by including the site as a stratifier, thus allowing the baseline hazard to depend on the site. Aβ(1‐38), Aβ(1‐40), and Aβ(1‐42) quartile limits were computed to visualize the temporal trajectory of participants with high to low biomarker levels.

As complementary analyses/robustness checks of the Cox regression, MLR analyses on the CDR‐SB were conducted as outlined in Section 2.5.1 in sample 2, thus including the same adjustments as in the MLR for MMSE and PACC analyses.

## 3 RESULTS

### 3.1 Participant characteristics

**FIGURE 2:**
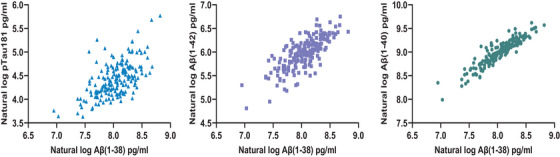
Correlation of CSF biomarkers with Aβ(1‐38). Correlations of CSF Aβ(1‐40), Aβ(1‐42), and p‐tau181 with Aβ(1‐38). Biomarkers are presented as their natural log transformations. Aβ(1‐38), amyloid beta 1‐38; CSF, cerebrospinal fluid; p‐tau181, tau protein phosphorylated at threonine 181.

In total, 177 SCD, MCI, and AD dementia participants were included in the study, with a mean follow‐up time of 1.54 years (SD = 1.42). The sample comprised 90 female (51%) and 87 male (49%) participants. On average, participants were 74.45 years of age (SD = 5.72; min: 61, max: 90) at baseline. Baseline demographics per diagnostic group can be inspected in Table 1. As depicted in Figure 2, the CSF biomarkers, particularly Aβ(1‐40), correlated highly with Aβ(1‐38) [(Aβ(1‐40): *r* = 0.92, *p* < 0.001; Aβ(1‐42): 0.75, *p* < 0.001; p‐tau181: 0.61, *p* < 0.001)].

**TABLE 1:** Participant characteristics per diagnostic group.

Characteristic	SCD (*n* = 56)	MCI (*n* = 60)	Dementia (*n* = 61)	Sample 1 Total (*N* = 177)	Sample 2 Total (*N* = 116)
Sex, *n* (%)					
Women	22 (39.3)	29 (48.3)	39 (63.9)	90 (50.8)	51 (44)
Men	34 (60.7)	31 (51.7)	22 (36.1)	87 (49.2)	65 (56)
Age, mean (SD), years	74.13 (4.84)	73.52 (5.59)	75.67 (6.42)	74.45 (5.72)	73.81 (5.23)
Education mean (SD), years	14.96 (3.08)	13.65 (3.12)	13.02 (2.98)	13.85 (3.15)	14.28 (3.16)
Converters^a^	3	23	–	–	26
CDR‐SB, mean (SD)^b^	0.48 (0.62)	1.67 (1.20)	–	–	1.09 (1.13)
MMSE, mean (SD)	29.18 (1.08)	27.08 (1.99)	22.98 (3.03)	26.33 (3.39)	28.09 (1.92)
PACC, mean (SD)	−0.30 (0.65)	−1.78 (1.00)	−3.54 (1.14)	−1.64 (1.55)	−1.05 (1.12)
Aβ(1‐38)	8.07 (0.29)	8.05 (0.29)	7.99 (0.35)	8.04 (0.31)	8.06 (0.29)
Aβ(1‐40)	9.02 (0.25)	9.00 (0.28)	8.05 (0.35)	9.00 (0.28)	9.01 (0.26)
Aβ(1‐42)	6.07 (0.33)	5.96 (0.32)	5.90 (0.35)	5.97 (0.34)	6.01 (0.33)
p‐tau181	4.31 (0.32)	4.45 (0.37)	4.53 (0.40)	4.43 (0.37)	4.38 (0.35)
p‐tau181/Aβ(1‐42)	1.76 (0.35)	1.52 (0.36)	1.37 (0.40)	1.54 (0.41)	1.64 (0.38)
Days since BL, mean (SD)	632.87 (547.78)	639.65 (583.27)	480.29 (469.13)	590.89 (543.12)	–
Follow‐ups, mean (SD)	1.69 (1.47)	1.66 (1.49)	1.23 (1.20)	1.54 (1.42)	–

*Note*: This table summarizes the cohort characteristics. In sample 1, a total of 177 biomarker‐positive participants from the DELCODE cohort were included and assigned to the SCD (*n* = 56), MCI (*n* = 60), or dementia (*n* = 61) groups. In sample 2 (*N* = 116), only the SCD (*n* = 56) and MCI (*n* = 60) groups were included. Demographics are presented and left blank to underline variables and groups included in sample 1 and sample 2, respectively. Biomarkers were naturally log‐transformed.

Abbreviation: Aβ(1‐38), amyloid beta 1‐38; BL, baseline; CDR‐SB, Clinical Dementia Rating Sum of the Boxes; MCI, mild cognitive impairment; MMSE, Mini‐Mental State Examination; PACC, Preclinical Alzheimer's Cognitive Composite; p‐tau181, tau protein phosphorylated at threonine 181; SCD, subjective cognitive decline.

^a^ Cox regression analyses comprised *n* = 89 from *N* = 116 participants with available conversion data.

^b^ MLR was performed on *n* = 113 from *N* = 116 participants with available CDR‐SB scores.

### 3.2 Sample 1: Aβ(1‐38) and longitudinal cognitive functioning

MLR models were performed to investigate the association of Aβ(1‐38) and longitudinal cognitive functioning. As described under 2.4.1.1, the comprehensive research approach included primarily linear models, which entail days since baseline as a time variable and are therefore more thoroughly reported in the following. Nevertheless, we also inform on the results of the LR tests, which compared both the initial and final linear against nonlinear models, that is, models with time represented as visit number (categorical predictor using dummy coding) instead of as days since baseline. Tables depicting the nonlinear models can be found in the Supplementary Materials.

#### 3.2.1 PACC

##### M1

**FIGURE 3:**
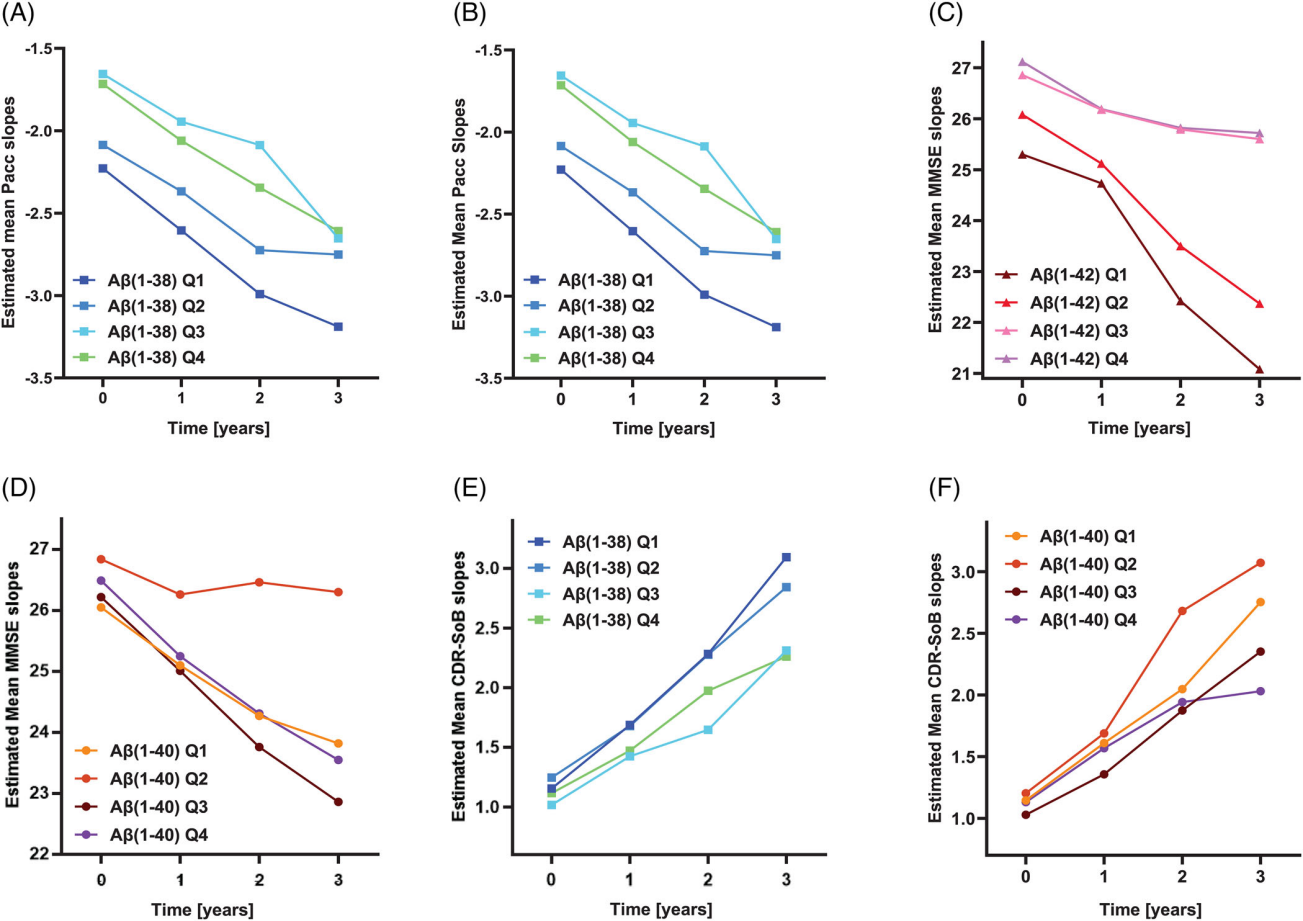
Longitudinal cognitive change at different CSF Aβ(1‐38), Aβ(1‐42), and Aβ(1‐40) baseline levels. Mean PACC, MMSE, and CDR‐SB score against time (0 = baseline, 1–3 = follow‐up visits) among Aβ‐positive participants with SCD, MCI, and AD in the DELCODE cohort. Focusing on linear models: the final results for M1 and M2 are presented for each outcome variable. Specifically, plots A, C, and E illustrate the results from M1 adjustments, whereas plots B, D, and F display the results from M2 adjustments. A, B, and F: by CSF Aβ(1‐38), C: Aβ(1‐42), D and F: Aβ(1‐40) baseline levels. Aβ(1‐38) quartile limits were 6.95 to 7.85 for quartile 1 (Q1), 7.85–8.07 for quartile 2 (Q2), >8.07–8.26 for quartile 3 (Q3), and >8.26–9.00 for quartile 4 (Q4). For Aβ(1‐42), quartile limits were 4.81–5.71 for Q1, 5.71–6.03 for Q2, 6.03–6.19 for Q3, and 6.19–6.75 for Q4. For Aβ(1‐40), quartiles limits were 7.86–8.83 for Q1, 8.83–9.03 for Q2, 9.03–9.19 for Q3, and 9.19–9.81 for Q4. Trajectories were derived from MLR models for PACC, MMSE, and CDR‐SB score change from baseline, respectively. All models were adjusted for age, sex, years of education, and screening diagnosis, as well as for interactions with time and these covariates. AD, Alzheimer's disease; Aβ(1‐38), amyloid beta 1‐38; CDR‐SB, Clinical Dementia Rating Sum of Boxes; CSF, cerebrospinal fluid; M1, model 1; M2, model 2; MCI, mild cognitive impairment; MLR, mixed linear regression; MMSE, Mini‐Mental State Examination; PACC, Preclinical Alzheimer's Cognitive Composite; p‐tau181, tau protein phosphorylated at threonine 181; SCD, subjective cognitive decline.

Analyses comprised observations until follow‐up 3 and entailed 153 participants with available PACC follow‐up data computations on at least one measurement occasion (baseline or follow‐up). Following the mixed modeling procedure outlined in Section 2.4.1.1., the initial model was reduced where possible without loss of goodness of fit. The final model allowed for an unstructured covariance matrix of the repeated measures (i.e., no homogeneity over time points was assumed) and a linear time effect. As visible in Table 2, the fixed (predictor) part consisted of sex, age, education years, time (modeled as days since baseline), Aβ(1‐38), Aβ(1‐42), p‐tau181, and diagnosis, as well as interactions of diagnosis×time, Aβ(1‐38)×time, and p‐tau181×time. So, there is a significant association between Aβ(1‐38) baseline levels and PACC change over time (see Table 2). The time trajectories are illustrated in Figure 3A. The nonlinear model also showed interaction between Aβ(1‐38) and time (see Table S1). The final linear model included predictors different from the final nonlinear model (see Table S1). Hence, LR testing could be performed only by comparing both initial models. The n.s. chi‐square test supported the validity of the linear model: X^2^(18, *N* = 153) = 19.51, *p* > 0.05).

**TABLE 2:** MLR: PACC M1 and M2.

						95% Confidence interval	
Parameter	Β	SE	df	t	Sig.	Lower bound	Upper bound
**M1**							
Intercept	−8.09	2.14	143.86	−3.78	<0.001	−12.33	−3.86
SCD	2.88	0.21	139.00	13.59	<0.001	2.46	3.30
MCI	1.61	0.19	145.19	8.53	<0.001	1.24	1.98
AD^a^	0.00	0.00	.	.	.	.	.
Time^b^	−7.59E‐3	2.66E‐3	60.80	−2.85	0.006	−0.01	0.00
Sex	0.51	0.15	129.83	3.29	<0.001	0.20	0.81
Age	−0.01	0.01	131.64	−0.58	0.564	−0.03	0.02
Education, years	0.12	0.02	133.44	4.93	<0.001	0.07	0.17
Aβ(1‐38)	0.48	0.41	138.48	1.19	0.237	−0.32	1.29
Aβ(1‐42)	0.33	0.30	123.41	1.10	0.272	−0.26	0.93
p‐tau181	−0.57	0.27	139.78	−2.07	0.041	−1.11	−0.02
Time×SCD	1.08E‐3	0.33E‐3	92.29	3.28	<0.001	0.43E‐3	1.74E‐3
Time×MCI	0.45E‐3	0.33E‐3	95.81	1.36	0.176	−0.21E‐3	1.12E‐3
Time×AD^a^	0.00	0.00	.	.	.	.	.
Time×Aβ(1‐38)	1.35E‐3	0.46E‐3	62.98	2.95	<0.001	0.44E‐3	2.27E‐3
Time×pTau181	−1.11E‐3	0.45E‐3	68.13	−2.45	0.017	−1.99E‐3	−0.21E‐3
**M2**							
Intercept	−9.58	2.77	141.40	−3.45	<0.001	−15.06	−4.10
SCD	2.90	0.21	136.75	13.62	<0.001	2.48	3.32
MCI	1.63	0.19	144.53	8.57	<0.001	1.25	2.01
AD^a^	0.00	0.00	.	.	.	.	.
Time^b^	−0.01	2.66E‐3	60.37	−2.82	0.006	−0.01	−2.20E‐3
Sex	0.50	0.15	130.83	3.25	0.001	0.20	0.81
Age	−0.01	0.01	131.86	−0.60	0.553	−0.04	0.02
Education, years	0.12	0.02	134.00	4.85	<0.001	0.07	0.17
Aβ(1‐38)	0.10	0.60	130.43	0.17	0.867	−1.09	1.29
Aβ(1‐40)	0.60	0.71	125.68	0.85	0.396	−0.80	2.00
Aβ(1‐42)	0.22	0.33	119.64	0.68	0.497	−0.43	0.88
p‐tau181	−0.61	0.28	140.81	−2.17	0.031	−1.16	−0.05
Time×SCD	1.08E‐3	0.33E‐3	91.92	3.27	0.002	0.42E‐3	1.74E‐3
Time×MCI	0.44E‐3	0.33E‐3	95.40	1.33	0.187	−0.22E‐3	1.11E‐3
Time×AD^a^	0.00	0.00	.	.	.	.	.
Time×Aβ(1‐38)	1.34E‐3	0.46E‐3	62.60	2.92	0.005	0.42E‐3	2.26E‐3
Time×p‐tau181	−1.09E‐3	0.45E‐3	67.78	−2.43	0.018	−1.98E‐3	−0.20E‐3

*Note*: MLR M1 and M2 with PACC as the dependent variable. In both models, the time trend was linear (i.e., including time as a continuous variable), and this linearity was validated using LR testing. LR tests were conducted to compare the initial linear models with their nonlinear (i.e., including time as a categorical variable) counterpart models, and when possible, final linear models were also tested against final nonlinear models. Biomarkers were naturally log‐transformed.

Abbreviations: AD, Alzheimer's disease; Aβ(1‐38), amyloid beta 1‐38; LR tests; Likelihood‐ratio tests; M1, model 1; M2, model 2; MCI, mild cognitive impairment; MLR, mixed linear regression; PACC, Preclinical Alzheimer's Cognitive Composite; p‐tau181, tau protein phosphorylated at threonine 181; SCD,  subjective cognitive decline.

^a^ Reference category.

^b^ Modeled as continuous variable, that is, days since baseline.

##### M2

Including Aβ(1‐40) beyond Aβ(1‐38) in the initial model resulted in a final model composed of time (modeled as days since baseline), sex, age, education years, Aβ(1‐38), Aβ(1‐40), Aβ(1‐42), p‐tau181, diagnosis, and interactions of diagnosis×time, Aβ(1‐38)×time, and p‐tau181×time, and thus showed a significant association of Aβ(1‐38) baseline levels with longitudinal PACC change over time (see Figure 3B and Table 2). By contrast, the nonlinear model did not identify such an association. Instead, an interaction of Aβ(1‐40) with time was found (see Table S1). As in M1, both final linear and nonlinear models differed in their fixed parts, thus restricting the comparison to the initial nonlinear and linear ones. The results were in favor of the linear model: X^2^(22, *N* = 153) = 25,57, *p* > 0.05.

#### 3.2.2 MMSE

##### M1

The same procedures as for the PACC score were applied. As said before, M1 was run twice, once with days since baseline as a continuous time variable (thus assuming a linear time effect), and once with visit number as a categorical time variable using dummy coding (thus allowing for a nonlinear time effect). The results of the final model can be found in Table 3. The final model consisted of demographics, Aβ(1‐38), Aβ(1‐42), p‐tau181, diagnosis, diagnosis×time, Aβ(1‐42)×time, and p‐tau181×time (see Table 3 and Figure 3C). However, Aβ(1‐42)×time only turned significant when removing Aβ(1‐38)×time. Of interest, when removing Aβ(1‐42)×time instead of Aβ(1‐38)×time, Aβ(1‐38)×time turned significant. For the nonlinear model, the same phenomenon occurred (i.e., removing interactions of Aβ(1‐38) or Aβ(1‐42) with time, respectively, resulting in the other one becoming significant). Again, the final linear and nonlinear models differed in their fixed parts. Hence, only their initial models were LR tested and the result supported the validity of the linear model: X^2^(18, *N* = 177) = 20.73, *p* > 0.05.

**TABLE 3:** MLR: MMSE M1 and M2.

						95% Confidence interval	
Parameter	Β	SE	df	t	Sig.	Lower bound	Upper bound
**M1**							
Intercept	20.16	4.52	150.52	4.46	<0.001	11.22	29.10
SCD	5.41	0.44	110.73	12.34	<0.001	4.54	6.28
MCI	3.80	0.39	165.36	9.78	<0.001	3.03	4.57
AD^a^	0.00	0.00	.	.	.	.	.
Time^b^	−0.01	0.01	119.72	−1.28	0.034	−0.02	3.90E‐3
Sex	0.66	0.34	166.11	1.97	0.051	−2.34E‐3	1.33
Age	−0.03	0.03	161.95	−0.96	0.327	−0.08	0.03
Education, years	0.21	0.05	162.71	4.05	<0.001	0.11	0.32
Aβ(1‐38)	1.29	0.91	165.93	1.42	0.243	−0.50	3.08
Aβ(1‐42)	−0.43	0.70	157.10	−0.62	0.679	−1.82	0.95
Ptau181	−1.32	0.58	151.14	−2.28	0.034	−2.47	−0.18
Time×SCD	3.58E‐3	0.83E‐3	128.38	4.31	<0.001	1.94E‐3	0.01
Time×MCI	2.60E‐3	0.77E‐3	124.55	3.40	<0.001	1.08E‐3	4.11E‐3
Time×AD^*^	0.00	0.00	.	.	.	.	.
Time×Aβ(1‐42)	3.16E‐3	0.97E‐3	110.22	3.27	<0.001	1.25E‐3	0.01
Time×Ptau181	−3.79E‐3	0.97E‐3	132.77	−3.90	<0.001	−0.01	−1.87E‐3
**M2**							
Intercept	14.17	6.09	165.17	2.33	0.021	2.14	26.20
SCD	5.46	0.43	165.07	12.56	<0.001	4.60	6.32
MCI	3.84	0.39	165.37	9.91	<0.001	3.08	4.61
AD^a^	0.00	0.00	.	.	.	.	.
Time^b^	−0.03	0.01	121.31	−2.89	0.005	−0.05	−0.01
Sex	0.66	0.34	165.45	1.96	0.052	−0.01	1.32
Age	−0.02	0.03	167.43	−0.90	0.367	−0.08	0.03
Education, years	0.22	0.05	161.93	4.12	<0.001	0.11	0.32
Aβ(1‐38)	−0.42	1.28	165.39	−0.33	0.742	−2.94	2.10
Aβ(1‐42)	−0.76	0.75	163.28	−1.02	0.310	−2.24	0.72
Aβ(1‐40)	2.44	1.51	165.11	1.62	0.107	−0.54	5.42
Ptau181	−1.43	0.59	168.90	−2.41	0.017	−2.59	−0.26
Time×SCD	3.57E‐3	0.81E‐3	127.56	4.41	<0.001	1.97E‐3	0.01
Time×MCI	2.56E‐3	0.75E‐3	124.11	3.40	<0.001	1.07E‐3	4.05E‐3
Time×AD^*^	0.00	0.00	.	.	.	.	.
Time×Aβ(1‐40)	0.01	1.51E‐3	122.82	3.68	<0.001	2.57E‐3	0.01
Time×Ptau181	−0.01	1.23E‐3	134.54	−4.60	<0.001	−0.01	−0.32E‐3

*Note*: MLR M1 and M2 with MMSE as the dependent variable. In both models, the time trend was linear (i.e., including time as a continuous variable), and this linearity was validated using LR testing. LR tests were conducted to compare the initial linear models with their nonlinear (i.e., including time as a categorical variable) counterpart models, and when possible, final linear models were also tested against final nonlinear models. Biomarkers were naturally log‐transformed.

Abbreviation: AD, Alzheimer's disease; Aβ(1‐38), amyloid beta 1‐38; LR tests, Likelihood‐ratio tests; M1, model 1; M2,  model 2; MCI, mild cognitive impairment; MLR, mixed linear regression; MMSE, Mini‐Mental State Examination; p‐tau181, tau protein phosphorylated at threonine 181; SCD,  subjective cognitive decline.

^a^ Reference category.

^b^ Modeled as continuous variable, that is, days since baseline.

##### M2

Due to a Hessian warning, the random intercept was removed from the final model. Furthermore, including Aβ(1‐40) into the initial model resulted in a final model, entailing demographics, Aβ(1‐38), Aβ(1‐40), Aβ(1‐42), diagnosis, time×diagnosis, time×Aβ(1‐40), and time×p‐tau181 (see Table 3). Again, Aβ(1‐38)×time had to be removed from the model due to its *p*‐value being far from significant. Instead, this final model showed a positive interaction of Aβ(1‐40) baseline levels and time (see Figure 3D). The final nonlinear model entailed the same fixed effects and likewise showed a significant Aβ(1‐40)×time interaction, but no association of Aβ(1‐38)×time with MMSE (see Table S2). LR testing of the initial models [X^2^(20, *N* = 177) = 24,63, *p* > 0.05] as well as of the final models [X^2^(10, *N* = 177) = 13.59, *p* > 0.05] favored the linear model.

### 3.3 Sample 2: Aβ(1‐38) and dementia conversion risk

#### 3.3.1 Cox regression

**FIGURE 4 alz14545-fig-0004:**
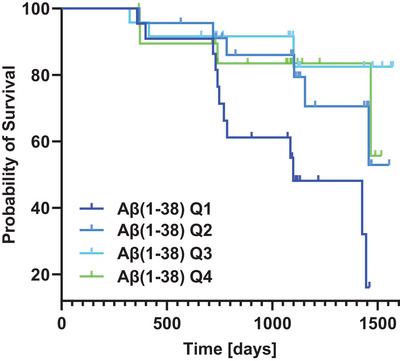
Kaplan–Meyer curves: Risk of AD conversion by CSF Aβ(1‐38) baseline levels. *N* = 89 (SCD = 46, MCI = 43); on the X‐axis the survival duration is given in days since baseline. The interval is terminated once the event occurred. The Y‐axis depicts the cumulative probability of conversion to AD dementia at a given time by CSF Aβ(1‐38) baseline levels. Aβ(1‐38) concentrations are depicted per natural log transformed quartile levels. Quartile limits were: quartile 1 (Q1) = 7.85, quartile 2 (Q2) = 8.07, quartile 3 (Q3) = 8.27 and quartile 4 (Q4) = 8.82. Minimum: 7.37, range 1.45, mean = 8.06, standard deviation = 029. Censorings were indicated by the black tick mark on the curves. AD, Alzheimer's disease; Aβ(1‐38), amyloid beta 1‐38; CSF, cerebrospinal fluid; MCI, mild cognitive impairment; SCD, subjective cognitive decline.

Of all 116 participants of sample 2, a total of 89 participants (SCD = 46; MCI = 39), 51 male and 38 female, provided conversion data, and 26 of these participants converted to an AD dementia (SCD = 3, MCI = 23). Among these 26 converters, sex was equally distributed, that is, 13 women and 13 men conversed. Crosstabs and Kaplan–Meyer plots showed a strong relation between diagnosis and conversion, as well as a significant association between Aβ(1‐38) baseline levels and conversion to dementia, as depicted in Figure 4. For the Cox regression, beyond the four markers—that is, Aβ(1‐38), Aβ(1‐40), Aβ(1‐42), and p‐tau181—all models for conversion risk were adjusted for by age, sex, education years, and screening diagnosis and stratified on testing site. The initial models (i.e., M1 and M2) are depicted in the flowchart (see Figure 1). The model was stepwise reduced by its least significant predictor. In M1, the final model included diagnosis, age, sex, education, Aβ(1‐38) (Exp(B) = 8.53E‐3, 95% confidence interval [CI]: 32.56E‐5‐0.22, *β = ‐4,76*, *p* = 0.004), and p‐tau181, as well as a time‐varying effect (i.e., interaction with time) of screening diagnosis, see Table S3. In view of the extreme HR of Aß(1‐38), i.e., 8.53E‐3, the analysis was repeated without stratification on site, because the number of sites (i.e., 10) was large relative to the sample size and it is known from statistical literature that adding predictors uncorrelated with the predictors already in the model moves the regression weights of the old predictors away from zero in generalized linear regression models such as logistic and Cox regression (Please include reference here: reference: Robinson LD, Jewell NP (1991). Some surprising results about covariate adjustment in logistic regression models. International Statistical Review, 58(2), 227‐240). This re‐analysis confirmed the negative relation of Aß(1‐38), and the positive relation of p‐tau181, with conversion, but gave less extreme effects, specifically a HR of 0.043 (with SE = 1.20, p = 0.008) instead of 8.53E‐3 for Aß(1‐38). Similarly, in M2, Aβ(1‐40) was removed immediately from the model due to its *p*‐value being far from significant. Thus the final model was equivalent to M1, which is depicted in Table S3, in terms of predictors included, including diagnosis, age, sex, education, Aβ(1‐38), and p‐tau181, showing a negative relation between higher Aβ(1‐38) baseline levels and the risk of conversion to AD dementia.

#### 3.3.2 MLR: Aβ(1‐38) and CDR‐SB

##### M1

The same procedures[Fig alz14545-fig-0004] as for the PACC and MMSE MLR were applied to the CDR‐SB scores, except that the sample now excluded the AD group that had already converted at baseline. Of all 116 participants, 113 had available CDR‐SB scores at baseline. The reported final model contained an unstructured covariance matrix of the repeated measures within centers, a random center effect and a linear time effect. As visible in Table 4, the fixed (predictor) part consisted of sex, age, education years, time (modeled as days since baseline), Aβ(1‐42), Aβ(1‐38), p‐tau181, diagnosis, as well as interactions of diagnosis×time, sex×time, age×time, Aβ(1‐38)×time, and p‐tau181×time, and showed a significant negative Aβ(1‐38)×time interaction (see Table 4 and Figure 3E). The nonlinear model resulted in the same final model and showed significant interactions of Aβ(1‐38) with time at follow‐ups 2 and 3 (see Table S4). LR testing of the initial linear versus initial nonlinear models [X^2^(16, *N* = 113) = 16,51, *p* > 0.05] as well as of the final linear versus nonlinear models [X^2^(12, *N* = 113) = ‐13.46, *p* > 0.05] supported the validity of the linear model.

**TABLE 4 alz14545-tbl-0004:** MLR: CDR‐SB M1 and M2.

						95% Confidence interval	
Parameter	Β	SE	df	T	Sig.	Lower bound	Upper bound
**M1**							
Intercept	1.33	2.94	107.07	0.45	0.652	−4.50	7.16
SCD	−1.21	0.19	104.21	−6.27	<0.001	−1.60	−0.83
MCI^a^	0.00	0.00	.	.	.	.	.
Time^b^	3.74E‐3	0.01	88.45	0.60	0.547	−0.01	0.02
Sex	−0.07	0.19	102.01	−0.35	0.724	−0.45	0.32
Age	0.02	0.02	103.75	0.88	0.379	−0.02	0.05
Education, years	−0.02	0.03	102.82	−0.80	0.425	−0.09	0.04
Aβ(1‐38)	−0.27	0.53	108.85	−0.51	0.608	−1.33	0.78
Aβ(1‐42)	−0.03	0.39	101.68	−0.09	0.932	−0.81	0.74
p‐tau181	0.47	0.36	109.74	1.31	0.192	−0.24	1.18
Time×SCD	−1.06E‐3	0.41E‐3	84.44	−2.61	0.011	−0.19E‐	−0.25E‐3
Time×MCI^*^	0.00	0.00	.	.	.	.	.
Time×sex	0.76E‐3	0.41E‐3	81.74	1.88	0.064	−0.05E‐3	1.57E‐3
Time×age	0.07E‐3	0.04E‐3	103.80	1.95	0.054	−0.00E‐3	0.15E‐3
Time×Aβ(1‐38)	−3.08E‐3	0.94E‐3	89.84	−3.27	0.002	−4.95E‐3	−1.21E‐3
Time×p‐tau181	3.90E‐3	0.83E‐3	95.45	4.72	<0.001	2.26E‐3	0.01
**M2**							
Intercept	0.85	3.59	104.16	0.24	0.813	−6.27	7.98
SCD	−1.21	0.19	103.39	−6.25	<0.001	−1.59	−0.83
MCI^a^	0.00	0.00	.	.	.	.	.
Time^b^	0.01	0.01	86.34	1.28	0.204	−0.01	0.02
Sex	−0.07	0.20	101.23	−0.37	0.709	−0.46	0.31
Age	0.01	0.02	102.54	0.85	0.398	−0.02	0.05
Education years	−0.02	0.03	101.75	−0.80	0.425	−0.09	0.04
Aβ(1‐38)	−0.43	0.79	103.04	−0.55	0.585	−1.99	1.13
Aβ(1‐40)	0.23	0.88	97.78	0.27	0.791	−1.51	1.97
Aβ(1‐42)	−0.08	0.42	100.49	−0.18	0.854	−0.90	0.75
p‐tau181	0.46	0.36	110.11	1.26	0.210	−0.26	1.17
Time×SCD	−1.07E‐3	0.41E‐3	85.37	−2.59	0.011	−1.89E‐3	−0.25E‐3
Time×MCI^*^	0.00	0.00	.	.	.	.	.
Time×sex	0.87E‐3	0.42E‐3	83.28	2.08	0.040	0.04E‐3	1.69E‐3
Time×age	0.08E‐3	0.04E‐3	106.53	2.01	0.047	0.00E‐3	0.15E‐3
Time×Aβ(1‐40)	−3.47E‐3	1.03E‐3	88.02	−3.36	0.001	−0.01	−1.42E‐3
Time×p‐tau181	3.95E‐3	0.82E‐3	94.17	4.79	<0.001	2.32E‐3	0.01

*Note*: MLR M1 and M2 with CDR‐SB as the dependent variable. In both models, the time trend was linear (i.e., including time as a continuous variable), and this linearity was validated using LR testing. LR tests were conducted to compare the initial linear models with their nonlinear (i.e., including time as a categorical variable) counterpart models, and when possible, final linear models were also tested against final nonlinear models. Biomarkers were naturally log‐transformed.

Abbreviation: AD, Alzheimer's disease; Aβ(1‐38), amyloid beta 1‐38; CDR‐SB, Clinical Dementia Rating Sum of Boxes; LR tests, Likelihood‐ratio tests; M1, model 1; M2, model 2; MCI, mild cognitive impairment; MLR, mixed linear regression; p‐tau181, tau protein phosphorylated at threonine 181; SCD, subjective cognitive decline.

^a^ Reference category.

^b^ Modeled as continuous variable, that is, days since baseline.

##### M2

The final model differed from M1 in replacing the interaction of Aβ(1‐38)×time with the interaction of Aβ(1‐40)×time (and including Aβ40 of course). Thus, including Aβ(1‐40) beyond Aβ(1‐38) resulted in the removal of Aβ(1‐38)×time from the model due to its higher *p*‐value as compared to the *p*‐value of Aβ(1‐40)×time. Of interest, when Aβ(1‐38)×time was removed from the model, the Aβ(1‐40)×time interaction turned significant (see Figure 3F). Likewise, when removing Aβ(1‐40)×time instead of Aβ(1‐38)×time, the latter turned significant (i.e., *p* = 0.002). In contrast, the final nonlinear M2 model included Aβ(1‐38)×time and not Aβ(1‐40)×time, as well as interactions of diagnosis×time, sex×time, and p‐tau181×time (see Table S4). The final linear and nonlinear models had different fixed parts, limiting the comparison to the initial nonlinear and linear models and favoring the validity of the linear model [X^2^(18, *N* = 113) = 17,31, *p* > 0.05].

## 4 DISCUSSION

The present study aimed to extend the knowledge on the role of CSF Aβ(1‐38) in AD‐related decline by replicating[Table alz14545-tbl-0004] previous findings^8^ first and introducing a unique methodological approach. Specifically, we aimed to assess which combination of key predictors, including Aβ(1‐38), best predicts cognitive change in individuals along the AD spectrum using LR testing. We also investigated whether higher CSF Aβ(1‐38) levels might be associated with a lower risk of converting to AD dementia. Unlike previous studies, we controlled for baseline diagnostic status as well as for baseline CSF Aβ(1‐40), that is, M2.

The Cox regression models (i.e., M1 and M2) yielded a decreased AD dementia conversion risk for individuals with higher baseline Aβ(1‐38) levels within 3 years. Furthermore, higher Aβ(1‐38) baseline levels were associated with slower PACC decline in M1 and M2, as well as slower CDR‐SB decline in M1. Including Aβ(1‐40) beyond Aβ(1‐38) in the model (i.e., M2) showed no relation between Aβ(1‐38) and CDR‐SB change. Overall, no association between Aβ(1‐38) and MMSE performance over time was found (i.e., neither in M1 nor in M2).

These mixed findings should be interpreted considering previous research results, such as those outlined in the following. First, PACC and MMSE score changes are frequently applied as behavioral measures of cognitive performance in AD research. Similar to almost identical studies using PACC or MMSE as outcome variables, such as Cullen et al.,^8^ the present study results on MMSE and PACC are in part opposing each other. Notably, although the MMSE is a frequently applied, quick screening tool in memory clinics and clinical trials, the PACC score is more powerful than the MMSE in measuring AD progression.^26^, ^27^ Specifically, acquiring a PACC score requires extensive neuropsychological testing involving administering various cognitive tests. Even more so, the PACC was based on a selection of tests particularly sensitive to amyloid‐related decline.^22^ The MMSE, on the other hand, is a quick screening tool for cognitive deficits that has been shown to produce less reliable and valid results than other dementia screening methods used in geriatric medicine.^26^ Thus the PACC score is more reliable than the MMSE in detecting cognitive change. Considering this, the lack of association between Aβ(1‐38) and MMSE score change in the MLR analyses might reflect a type II error.

Second, our statistical approach shows that in some MLR models the association depended on whether we adjusted for Aβ(1‐40). Notably, removing either Aβ(1‐38) or Aβ(1‐40) from the model led to significant results in one or the other. The association also depended on whether the time effect was assumed to be linear or nonlinear (see Supplementary Materials). Furthermore, Aβ(1‐38) and Aβ(1‐40) were highly correlated, which raised the question of whether Aβ(1‐38) might deliver additional predictive value to Aβ(1‐40) after all. Moreover, the fact that our results consistently show that higher p‐tau181 is associated with stronger AD‐related decline, which is in line with previous studies,^28^, ^29^ may suggest that the relations of Aβ(1‐38), Aβ(1‐40), and Aβ(1‐42) with AD‐related decline may depend on whether we adjust for p‐tau181.

Third, clinical and preclinical studies suggest beneficial associations between higher levels of the short Aβ(1‐38) peptides and AD pathology, such as inhibition of Aβ(1‐42) aggregation in vitro^10^, ^30^ and slower cognitive decline in humans in vivo.^8^, ^31^ In line with this previous research, our findings suggest that perhaps there is a beneficial association between higher Aβ (1‐38) levels and AD‐related decline, offering hope for future research and treatment of AD.

Hypothetically, the link between Aβ(1‐38) and AD‐related decline may point toward previously unknown disease mechanisms. For instance, reduced Aβ(1‐38) concentrations in the CSF may reflect a breakdown in Aβ degradation. In vitro experiments have shown that human CSF Aβ(1‐34) peptides are beta‐site of amyloid precursor protein (APP) cleaving enzyme 1 (BACE1)‐derived degradation intermediates related to amyloid clearance and the later progression to AD dementia.^32^, ^33^ This suggests that BACE1 expression may not only result in amyloidogenic Aβ production but can lead to different degradation pathways.^32^ For example, Aβ degradation results in the shorter favorable Aβ(1‐34) species in human cell samples when sufficient or excess BACE1 levels are present.^32^, ^34^ Moreover, BACE1 inhibitors have frequently failed to achieve efficacy and safety in Phase I and II trials.^35^, ^36^, ^37^ Possibly, BACE1 inhibitors prevent the degradation of long Aβ(1‐42) into shorter, more beneficial forms, thereby leading to worsening or absence of health improvement in patients with AD. A similar unknown mechanism might also apply to the Aβ(1‐38) degradation pathway. Following these previous lines, understanding such a hypothetical unknown mechanism might be worthwhile, as it may ultimately shape the development of effective treatments—for example, improving γ‐secretase modulators as potential targets for drug development.

In conclusion, the present findings may suggest that perhaps higher Aβ(1‐38) CSF concentrations may have a beneficial impact on AD‐related decline, which could inform individual risk prediction in memory clinic patients. In addition, based on the current state of research, the role of short Aβ peptides in AD may have been previously overlooked. Therefore, an expansion of research on this topic, including the investigation of Aβ(1‐34), Aβ(1‐36), Aβ(1‐37), and Aβ(1‐39), is highly warranted.

Investigating Aβ ratios, for example, may offer additional valuable insights, especially concerning the mechanisms of APP cleavage. Although Aβ(1‐42) is a well‐established biomarker for AD, previous research has shown that measuring CSF Aβ ratios, such as Aβ(1‐42/1‐40) and Aβ(1‐42/1‐38), provides better diagnostic accuracy compared to assessing Aβ(1‐42) alone.^38^ Recent findings indicate that the Aβ(1‐42/1‐37) ratio has outperformed the Aβ(1‐42/1‐40) ratio in distinguishing AD from healthy individuals.^39^ Furthermore, a study examining the Aβ(1‐42/1‐38) ratio in cognitively unimpaired older adults found that lower Aβ(1‐42/1‐38) ratios, reflecting higher levels of Aβ(1‐38), were linked to slower cognitive decline,^31^ which confirms a potential relationship of Aβ(1‐38) and cognitive decline. Our current study focused on the individual Aβ(1‐38) species and predicting cognitive decline rather than on diagnostic accuracy and Aβ(1‐38) ratios; therefore, we strongly recommend exploring Aβ(1‐38) ratios as a vital avenue for future research.

In addition, recent research indicates that p‐tau217 may be specific to AD^40^ and could outperform p‐tau181 as a diagnostic biomarker for AD.^41^ Consequently, it would have been valuable to explore whether the inclusion of p‐tau181 versus p‐tau217 would yield different model outcomes. However, p‐tau217 has yet to be measured in the DELCODE cohort, which restricted our ability to conduct this comparison but offers an opportunity for future research.

Missing data limited the number of follow‐up observations that could be included in our statistical analyses. Specifically, missing data as of follow‐up 4 and participant attrition, especially in the AD group, limited the inclusion of additional follow‐up data and, therefore, the statistical examination beyond the 4 years. Nevertheless, our MLR and Cox regression model analyses consistently showed that screening diagnosis and, in part, Aβ(1‐40) significantly predict longitudinal cognitive change and AD conversion risk. Our study controlled for these major confounding factors. Moreover, the present research possesses a large sample size; entailed in‐depth neuropsychometric assessments; used longitudinal data; included well‐characterized SCD, MCI, and AD groups (i.e., preclinical to clinical stages of dementia); and provided a clear biological and clinical definition of AD. As such, the decision to include only participants with a positive Aβ(1‐42)/p‐tau181 ratio can be seen as a strength of the current study, even though this led to the exclusion of 212 participants. Indeed, the Aβ(1‐42)/p‐tau181 ratio has been shown to reliably select individuals along the AD spectrum.^42^, ^43^ Nevertheless, comparing amyloid‐negative individuals might provide additional valuable insights into the role of short Aβ peptides in cognitive decline in individuals outside the AD spectrum. However, this investigation was beyond the scope of the present study. We recommend that future studies strive to explore potential differences between the impact of Aβ(1‐38) on biomarker‐negative individuals, cognitively unimpaired individuals, and those along the AD continuum. This may help to clarify the specific role of Aβ(1‐38) in AD.

Finally, our study was performed in a German cohort, and therefore, Western, educated, industrialized, rich and democratic (WEIRD) individuals are overrepresented in the sample. As a result, the present findings may generalize to WEIRD populations only. Future studies should aim to expand the investigation on Aβ(1‐38) to non‐WEIRD populations.

## CONFLICT OF INTEREST STATEMENT

The authors have no conflicts of interest to declare that are relevant to the content of this article. Author disclosures are available in the Supporting Information.

## ETHICS STATEMENT

The respective centers’ local ethics committees (e.g., Charité – Universitätsmedizin Berlin) approved the study. Following the 1947 Nuremberg Code, the 1964 Helsinki Declaration, and the Economic and Social Research Council (ESRC), participants were fully briefed on the purpose of the research project, methods, and intended outcomes, including any potential risks associated with their participation. Standard procedures for incidental findings in CSF were established. Participation could be withdrawn at any time. All information provided by participants was treated confidentially and processed anonymously (i.e., use of pseudonymization), and commercial use is precluded. A web‐based electronic case report form (eCRF; i.e., web‐spirit, 2mt software) was used to store research data.^17^ Data handling and quality, including plausibility and data source verification through onsite monitoring visits, were checked regularly upon data entry. The present study did not involve any participant risk because data had already been gathered. Detailed recruitment and accessibility procedures as well as the first DELCODE baseline data are discussed extensively elsewhere.^17^

## CONSENT STATEMENT

All participants provided written informed consent and approved anonymous publication of this information.

## Supporting information



Supporting Information

Supporting Information
