# Supplementary material for: Linking higher amyloid beta 1‐38 (Aβ(1‐38)) levels to reduced Alzheimer's disease progression risk
Source: Alzheimers Dement. 2025 Jan 27;21(2):e14545. doi: 10.1002/alz.14545 (PMC11863357; doi:10.1002/alz.14545)
Supplement: Supplementary file 1 — Supporting Information [file ALZ-21-e14545-s002.docx]

**Supplementary Material**

**Table S1** MLR: Non-linear PACC M1 and M2

| Paramater | β | Std. Error | df | t | Sig. | 95% Confidence Interval | |
| --- | --- | --- | --- | --- | --- | --- | --- |
|  |  |  |  |  |  | Lower Bound | Upper Bound |
| **M1** |  |  |  |  |  |  |  |
| Intercept | -7.80 | 2.16 | 144.87 | -3.61 | <.001 | -12.07 | -3.54 |
| SCD | 2.89 | 0.21 | 141.48 | 13.47 | <.001 | 2.46 | 3.31 |
| MCI | 1.59 | 0.19 | 144.05 | 8.36 | <.001 | 1.21 | 1.97 |
| AD* | 0.00 | 0.00 | . | . | . | . | . |
| FU1 | -2.69 | 1.62 | 100.38 | -1.66 | .100 | -5.91 | 0.53 |
| FU2 | -4.96 | 2.47 | 82.14 | -2.01 | .048 | -9.88 | -0.05 |
| FU3 | -8.36 | 3.25 | 32.44 | -2.58 | .015 | -14.97 | -1.76 |
| BL* | 0.00 | 0.00 | . | . | . | . | . |
| Sex | 0.47 | 0.15 | 131.60 | 3.02 | .003 | 0.16 | 0.77 |
| Age | -0.01 | 0.01 | 134.02 | -0.75 | .456 | -0.04 | 0.02 |
| Education | 0.11 | 0.03 | 143.82 | 4.45 | <.001 | 0.06 | 0.16 |
| Aβ(1-38) | 0.52 | 0.41 | 140.52 | 1.27 | .205 | -0.29 | 1.33 |
| Aβ(1-42) | 0.31 | 0.30 | 126.26 | 1.04 | .302 | -0.29 | 0.91 |
| Ptau181 | -0.60 | 0.28 | 140.51 | -2.17 | .032 | -1.15 | -0.05 |
| FU1 x SCD | 0.51 | 0.18 | 103.02 | 2.80 | .006 | 0.15 | 0.87 |
| FU2 x SCD | 0.95 | 0.33 | 92.80 | 2.87 | .005 | 0.29 | 1.61 |
| FU3 x SCD | 1.16 | 0.49 | 32.59 | 2.36 | .024 | 0.16 | 2.15 |
| BL x SCD | 0.00 | 0.00 | . | . | . | . | . |
| FU1 x MCI | 0.19 | 0.18 | 104.31 | 1.06 | .293 | -0.16 | 0.54 |
| FU2 x MCI | 0.50 | 0.33 | 92.70 | 1.55 | .124 | -0.14 | 1.15 |
| FU3 x MCI | 0.24 | 0.49 | 33.17 | 0.49 | .626 | -0.76 | 1.24 |
| BL x MCI | 0.00 | 0.00 | . | . | . | . | . |
| FU1 x AD | 0.00 | 0.00 | . | . | . | . | . |
| FU2 x AD | 0.00 | 0.00 | . | . | . | . | . |
| FU3 x AD | 0.00 | 0.00 | . | . | . | . | . |
| BL x AD | 0.00 | 0.00 | . | . | . | . | . |
| FU1 x education | 0.01 | 0.02 | 99.68 | 0.36 | .722 | -0.03 | 0.04 |
| FU2 x education | -0.05 | 0.03 | 81.08 | -1.79 | .077 | -0.11 | 0.01 |
| FU3 x education | -0.05 | 0.04 | 32.38 | -1.28 | .209 | -0.13 | 0.03 |
| BL x education | 0.00 | 0.00 | . | . | . | . | . |
| FU1 x Aβ(1-38) | 0.35 | 0.27 | 99.08 | 1.31 | .193 | -0.18 | 0.89 |
| FU2 x Aβ(1-38) | 1.02 | 0.42 | 81.22 | 2.42 | .018 | 0.18 | 1.86 |
| FU3 x Aβ(1-38) | 1.52 | 0.57 | 33.77 | 2.67 | .011 | 0.36 | 2.67 |
| BL x Aβ(1-38) | 0.00 | 0.00 | . | . | . | . | . |
| FU1 x Ptau181 | -0.18 | 0.26 | 97.51 | -0.70 | .486 | -0.71 | 0.34 |
| FU2 x Ptau181 | -0.88 | 0.42 | 82.00 | -2.10 | .039 | -1.72 | -0.04 |
| FU3 x Ptau181 | -1.06 | 0.59 | 35.03 | -1.78 | .083 | -2.26 | 0.15 |
| BL x Ptau181 | 0.00 | 0.00 | . | . | . | . | . |
| **M2** |  |  |  |  |  |  |  |
| Intercept | -9.71 | 2.79 | 143.58 | -3.48 | <.001 | -15.23 | -4.19 |
| SCD | 2.91 | 0.21 | 139.65 | 13.54 | <.001 | 2.48 | 3.33 |
| MCI | 1.61 | 0.19 | 144.08 | 8.42 | <.001 | 1.23 | 1.99 |
| AD | 0.00 | 0.00 | . | . | . | . | . |
| FU1 | -1.87 | 2.03 | 101.20 | -0.92 | .359 | -5.89 | 2.15 |
| FU2 | -6.16 | 3.03 | 80.30 | -2.03 | .046 | -12.19 | -0.12 |
| FU3 | -11.17 | 4.18 | 38.43 | -2.67 | .011 | -19.62 | -2.71 |
| BL | 0.00 | 0.00 | . | . | . | . | . |
| Sex | 0.46 | 0.15 | 128.03 | 3.01 | .003 | 0.16 | 0.77 |
| Age | -0.01 | 0.01 | 131.69 | -0.79 | .430 | -0.04 | 0.02 |
| Education | 0.11 | 0.03 | 143.26 | 4.45 | <.001 | 0.06 | 0.16 |
| Aβ(1-38) | -0.01 | 0.60 | 125.72 | -0.02 | .987 | -1.19 | 1.18 |
| Aβ(1-40) | 0.78 | 0.71 | 130.47 | 1.10 | .272 | -0.62 | 2.18 |
| Aβ(1-42) | 0.21 | 0.33 | 116.02 | 0.63 | .529 | -0.44 | 0.86 |
| Ptau181 | -0.65 | 0.28 | 141.36 | -2.30 | .023 | -1.20 | -0.09 |
| FU1 x SCD | 0.55 | 0.18 | 102.38 | 3.06 | .003 | 0.19 | 0.90 |
| FU2 x SCD | 1.00 | 0.32 | 94.17 | 3.07 | .003 | 0.35 | 1.64 |
| FU3 x SCD | 1.27 | 0.49 | 35.92 | 2.62 | .013 | 0.29 | 2.26 |
| BL x SCD* | 0.00 | 0.00 | . | . | . | . | . |
| FU1 x MCI | 0.21 | 0.18 | 103.96 | 1.19 | .237 | -0.14 | 0.56 |
| FU2 x MCI | 0.55 | 0.32 | 94.26 | 1.71 | .091 | -0.09 | 1.18 |
| FU3 x MCI | 0.36 | 0.48 | 36.24 | 0.74 | .462 | -0.62 | 1.34 |
| BL x MCI* | 0.00 | 0.00 | . | . | . | . | . |
| FU1 x AD* | 0.00 | 0.00 | . | . | . | . | . |
| FU2 x AD* | 0.00 | 0.00 | . | . | . | . | . |
| FU3 x AD* | 0.00 | 0.00 | . | . | . | . | . |
| BL x AD* | 0.00 | 0.00 | . | . | . | . | . |
| FU1 x education | 0.01 | 0.02 | 99.64 | 0.29 | .772 | -0.03 | 0.04 |
| FU2 x education | -0.05 | 0.03 | 81.45 | -1.78 | .078 | -0.11 | 0.01 |
| FU3 x education | -0.05 | 0.04 | 37.17 | -1.26 | .215 | -0.13 | 0.03 |
| BL x education* | 0.00 | 0.00 | . | . | . | . | . |
| FU1 x Aβ(1-40) | 0.16 | 0.29 | 99.64 | 0.54 | .589 | -0.42 | 0.74 |
| FU2 x Aβ(1-40) | 1.03 | 0.45 | 79.62 | 2.30 | .024 | 0.14 | 1.91 |
| FU3 x Aβ(1-40) | 1.66 | 0.63 | 39.33 | 2.63 | .012 | 0.39 | 2.94 |
| BL x Aβ(1-40)* | 0.00 | 0.00 | . | . | . | . | . |
| FU1 x Ptau181 | -0.05 | 0.26 | 97.30 | -0.18 | .854 | -0.57 | 0.48 |
| FU2 x Ptau181 | -0.85 | 0.42 | 81.68 | -2.02 | .046 | -1.69 | -0.01 |
| FU3 x Ptau181 | -1.08 | 0.61 | 39.57 | -1.76 | .086 | -2.31 | 0.16 |
| BL x Ptau181* | 0.00 | 0.00 | . | . | . | . | . |

Abbreviations: MLR = mixed linear regression, M1 = model 1, M2 = model 2, SCD = subjective cognitive decline, MCI = mild cognitive impairment, AD = Alzheimer’s disease dementia, PACC = preclinical Alzheimer’s composite score, Aβ = β-amyloid, pTau181 = tau protein phosphorylated at threonine 181, BL = baseline, FU1/2/3 = follow-up one, two, and three respectively, LR-tests = Likelihood-ratio tests.

Note. MLR M1 and M2 with PACC as the dependent variable. In both models, the time trend was non-linear (i.e., including time as a categorical variable). LR-tests were conducted to compare the initial linear (i.e., including time as a continuous variable), models with their non-linear counterpart models, and when possible, final linear models were also tested against final non-linear models. Biomarkers were naturally log-transformed

*reference category

^†^modelled in visit numbers, using dummy coding with baseline visit as reference category.

**Table S2** MLR: Non-linear MMSE M1 and M2

| Parameter | β | Std. Error | Df | T | Sig. | 95% Confidence Interval | |
| --- | --- | --- | --- | --- | --- | --- | --- |
|  |  |  |  |  |  | Lower Bound | Upper Bound |
| **M1** |  |  |  |  |  |  |  |
| Intercept | 19.59 | 4.67 | 159.55 | 4.19 | <.001 | 10.36 | 28.81 |
| SCD | 5.40 | 0.45 | 118.63 | 11.88 | <.001 | 4.50 | 6.30 |
| MCI | 3.78 | 0.40 | 169.24 | 9.43 | <.001 | 2.99 | 4.58 |
| AD* | 0.00 | 0.00 | . | . | . | . | . |
| FU1 | -0.71 | 4.89 | 146.16 | -0.15 | .884 | -10.38 | 8.95 |
| FU2 | -13.11 | 7.58 | 130.64 | -1.73 | .086 | -28.11 | 1.89 |
| FU3 | -26.25 | 11.37 | 74.77 | -2.31 | .024 | -48.90 | -3.60 |
| BL* | 0.00 | 0.00 | . | . | . | . | . |
| Sex | 0.66 | 0.34 | 165.54 | 1.95 | .053 | -0.01 | 1.32 |
| Age | -0.03 | 0.03 | 163.29 | -1.13 | .262 | -0.09 | 0.02 |
| Edyears | 0.21 | 0.05 | 162.24 | 4.04 | <.001 | 0.11 | 0.32 |
| Aβ(1-38) | 1.26 | 0.94 | 168.15 | 1.33 | .185 | -0.61 | 3.12 |
| Aβ(1-42) | 0.12 | 0.73 | 160.66 | 0.16 | .870 | -1.32 | 1.56 |
| Ptau181 | -1.81 | 0.60 | 151.93 | -3.02 | .003 | -3.00 | -0.62 |
| FU1 x SCD | 1.26 | 0.49 | 145.32 | 2.58 | .011 | 0.29 | 2.22 |
| FU2 x SCD | 2.07 | 0.78 | 133.82 | 2.66 | .009 | 0.53 | 3.61 |
| FU3 x SCD | 4.42 | 1.16 | 73.37 | 3.83 | <.001 | 2.12 | 6.73 |
| BL x SCD* | 0.00 | 0.00 | . | . | . | . | . |
| FU1 x MCI | 0.86 | 0.46 | 146.20 | 1.89 | .061 | -0.04 | 1.76 |
| FU2 x MCI | 1.53 | 0.71 | 131.58 | 2.15 | .034 | 0.12 | 2.94 |
| FU3 x MCI | 3.26 | 1.06 | 74.15 | 3.06 | .003 | 1.14 | 5.38 |
| BL x MCI* | 0.00 | 0.00 | . | . | . | . | . |
| FU1 x AD* | 0.00 | 0.00 | . | . | . | . | . |
| FU2 x AD* | 0.00 | 0.00 | . | . | . | . | . |
| FU3 x AD* | 0.00 | 0.00 | . | . | . | . | . |
| BL x AD* | 0.00 | 0.00 | . | . | . | . | . |
| FU1 x Aβ(1-38) | 0.43 | 1.09 | 145.97 | 0.39 | .695 | -1.72 | 2.58 |
| FU2 x Aβ(1-38) | 2.39 | 1.72 | 132.58 | 1.40 | .165 | -1.00 | 5.79 |
| FU3 x Aβ(1-38) | 4.72 | 2.46 | 69.44 | 1.92 | .059 | -0.19 | 9.63 |
| BL x Aβ(1-38)* | 0.00 | 0.00 | . | . | . | . | . |
| FU1 x Aβ(1-42) | -0.64 | 0.83 | 145.50 | -0.77 | .441 | -2.27 | 0.99 |
| FU2 x Aβ(1-42) | 1.80 | 1.24 | 126.17 | 1.45 | .150 | -0.66 | 4.25 |
| FU3 x Aβ(1-42) | 2.07 | 1.74 | 67.88 | 1.19 | .239 | -1.41 | 5.54 |
| BL x Aβ(1-42)* | 0.00 | 0.00 | . | . | . | . | . |
| FU1 x Ptau181 | -0.13 | 0.70 | 146.46 | -0.18 | .855 | -1.51 | 1.26 |
| FU2 x Ptau181 | -4.62 | 1.13 | 135.29 | -4.08 | <.001 | -6.86 | -2.38 |
| FU3 x Ptau181 | -6.81 | 1.65 | 70.73 | -4.12 | <.001 | -10.11 | -3.52 |
| BL x Ptau181* | 0.00 | 0.00 | . | . | . | . | . |
| **M2** |  |  |  |  |  |  |  |
| Intercept | 12.04 | 6.27 | 172.49 | 1.92 | .056 | -0.33 | 24.41 |
| SCD | 5.49 | 0.45 | 170.75 | 12.25 | <.001 | 4.61 | 6.38 |
| MCI | 3.84 | 0.40 | 169.10 | 9.58 | <.001 | 3.05 | 4.63 |
| AD* | 0.00 | 0.00 | . | . | . | . | . |
| FU1 | -2.64 | 6.30 | 148.07 | -0.42 | .676 | -15.08 | 9.81 |
| FU2 | -31.10 | 9.45 | 129.28 | -3.29 | .001 | -49.80 | -12.39 |
| FU3 | -44.31 | 14.48 | 77.14 | -3.06 | .003 | -73.14 | -15.47 |
| BL* | 0.00 | 0.00 | . | . | . | . | . |
| Sex | 0.63 | 0.33 | 164.98 | 1.89 | .061 | -0.03 | 1.29 |
| Age | -0.03 | 0.03 | 167.52 | -1.07 | .285 | -0.08 | 0.02 |
| Education | 0.21 | 0.05 | 161.70 | 4.03 | <.001 | 0.11 | 0.32 |
| Aβ(1-38) | -0.37 | 1.28 | 165.00 | -0.29 | .775 | -2.89 | 2.15 |
| Aβ(1-42) | -0.74 | 0.75 | 162.99 | -0.99 | .323 | -2.22 | 0.74 |
| Aβ(1-40) | 2.94 | 1.52 | 170.73 | 1.94 | .054 | -0.06 | 5.94 |
| Ptau181 | -2.02 | 0.61 | 169.65 | -3.32 | .001 | -3.22 | -0.82 |
| FU1 x SCD | 1.14 | 0.47 | 147.03 | 2.41 | .017 | 0.20 | 2.08 |
| FU2 x SCD | 2.11 | 0.74 | 134.63 | 2.84 | .005 | 0.64 | 3.58 |
| FU3 x SCD | 4.73 | 1.14 | 75.83 | 4.17 | <.001 | 2.47 | 7.00 |
| BL x SCD* | 0.00 | 0.00 | . | . | . | . | . |
| FU1 x MCI | 0.84 | 0.45 | 148.18 | 1.87 | .064 | -0.05 | 1.74 |
| FU2 x MCI | 1.60 | 0.70 | 132.37 | 2.30 | .023 | 0.22 | 2.98 |
| FU3 x MCI | 3.49 | 1.07 | 76.81 | 3.26 | .002 | 1.36 | 5.61 |
| BL x MCI* | 0.00 | 0.00 | . | . | . | . | . |
| FU1 x AD* | 0.00 | 0.00 | . | . | . | . | . |
| FU2 x AD* | 0.00 | 0.00 | . | . | . | . | . |
| FU3 x AD* | 0.00 | 0.00 | . | . | . | . | . |
| BL x AD* | 0.00 | 0.00 | . | . | . | . | . |
| FU1 x Aβ(1-40) | 0.27 | 0.89 | 148.04 | 0.30 | .764 | -1.50 | 2.03 |
| FU2 x Aβ(1-40) | 5.89 | 1.37 | 132.13 | 4.29 | <.001 | 3.17 | 8.61 |
| FU3 x Aβ(1-40) | 8.04 | 2.09 | 75.64 | 3.85 | <.001 | 3.88 | 12.19 |
| BL x Aβ(1-40)* | 0.00 | 0.00 | . | . | . | . | . |
| FU1 x Ptau181 | -0.31 | 0.70 | 148.54 | -0.45 | .656 | -1.69 | 1.07 |
| FU2 x Ptau181 | -5.78 | 1.11 | 135.65 | -5.20 | <.001 | -7.98 | -3.58 |
| FU3 x Ptau181 | -7.78 | 1.74 | 77.85 | -4.48 | <.001 | -11.24 | -4.32 |
| BL x Ptau181* | 0.00 | 0.00 | . | . | . | . | . |

Abbreviations: MLR = mixed linear regression, M1 = model 1, M2 = model 2, SCD = subjective cognitive decline, MCI = mild cognitive impairment, AD = Alzheimer’s disease dementia, MMSE = Mini-Mental Status Test, Aβ = β-amyloid, pTau181 = tau protein phosphorylated at threonine 181, BL = baseline, FU1/2/3 = follow-up one, two, and three respectively, LR-tests = Likelihood-ratio tests.

Note. MLR M1 and M2 with MMSE as the dependent variable. In both models, the time trend was non-linear (i.e., including time as a categorical variable). LR-tests were conducted to compare the initial linear (i.e., including time as a continuous variable), models with their non-linear counterpart models, and when possible, final linear models were also tested against final non-linear models. Biomarkers were naturally log-transformed.

*reference category

^†^modelled in visit numbers, using dummy coding with baseline visit as reference category.

**Table S3** Cox regression in sample 2

| Parameter | B | SE | Wald | df | Sig. | Exp(B) | 95% CI for Exp(B) | |
| --- | --- | --- | --- | --- | --- | --- | --- | --- |
|  |  |  |  |  |  |  | Lower | Upper |
| Diagnosis | 6.22 | 3.05 | 4.17 | 1 | .041 | 504.59 | 1.29 | 197556.30 |
| Age | 0.12 | 0.07 | 3.13 | 1 | .077 | 1.13 | 0.99 | 1.30 |
| Sex | 0.28 | 0.50 | 0.31 | 1 | .575 | 1.32 | 0.50 | 3.51 |
| Education | -0.12 | 0.09 | 1.87 | 1 | .171 | 0.88 | 0.74 | 1.05 |
| Aβ(1-38) | -4.76 | 1.67 | 8.17 | 1 | .004 | 8.53E-3 | 0.00 | 0.22 |
| pTau181 | 3.16 | 1.45 | 4.73 | 1 | .030 | 23.56 | 1.37 | 406.07 |
| Time x diagnosis | -0.01 | 0.00 | 3.36 | 1 | .067 | 0.99 | 0.99 | 1.00 |

Abbreviations: Aβ = β-amyloid, pTau181 = tau protein phosphorylated at threonine 181

Note. The table shows the results of the Cox regression analyses conducted in sample 2 and stratified for the testing site. The model underwent stepwise reduction using the backward modelling method, i.e., removing the least significant predictor. Since M1 and M2 were found to be equivalent, only one model is presented. Biomarkers were naturally log-transformed.

**Table S4** MLR: Non-linear CDR-SoB M1 and M2 in sample 2

| Paramater | β | Std. Error | df | t | Sig. | 95% Confidence Interval | |
| --- | --- | --- | --- | --- | --- | --- | --- |
|  |  |  |  |  |  | Lower Bound | Upper Bound |
| **M1** |  |  |  |  |  |  |  |
| Intercept | 1.58 | 3.09 | 105.46 | 0.51 | .611 | -4.55 | 7.70 |
| SCD | -1.19 | 0.20 | 102.64 | -5.88 | <.001 | -1.59 | -0.79 |
| MCI* | 0.00 | 0.00 | . | . | . | . | . |
| FU1 | 1.30 | 4.35 | 89.96 | 0.30 | .766 | -7.34 | 9.94 |
| FU2 | 3.17 | 5.20 | 84.78 | 0.61 | .545 | -7.18 | 13.51 |
| FU3 | 8.83 | 8.44 | 72.83 | 1.05 | .299 | -8.00 | 25.66 |
| BL* | 0.00 | 0.00 | . | . | . | . | . |
| Sex | -0.18 | 0.20 | 104.53 | -0.90 | .371 | -0.59 | 0.22 |
| Age | 0.01 | 0.02 | 103.96 | 0.65 | .520 | -0.02 | 0.05 |
| Edyears | -0.03 | 0.03 | 103.23 | -0.98 | .332 | -0.09 | 0.03 |
| Aβ(1-38) | -0.19 | 0.55 | 111.87 | -0.35 | .724 | -1.29 | 0.90 |
| Aβ(1-42) | -0.05 | 0.40 | 102.31 | -0.14 | .892 | -0.84 | 0.73 |
| Ptau181 | 0.38 | 0.37 | 104.61 | 1.02 | .312 | -0.36 | 1.12 |
| FU1 x SCD | -0.44 | 0.28 | 90.99 | -1.57 | .121 | -1.01 | 0.12 |
| FU2 x SCD | -0.77 | 0.34 | 84.56 | -2.29 | .025 | -1.43 | -0.10 |
| FU3 x SCD | -1.13 | 0.53 | 69.83 | -2.15 | .035 | -2.19 | -0.08 |
| BL x SCD* | 0.00 | 0.00 | . | . | . | . | . |
| FU1 x MCI* | 0.00 | 0.00 | . | . | . | . | . |
| FU2 x MCI* | 0.00 | 0.00 | . | . | . | . | . |
| FU3 x MCI* | 0.00 | 0.00 | . | . | . | . | . |
| BL x MCI* | 0.00 | 0.00 | . | . | . | . | . |
| FU1 x sex | 0.51 | 0.28 | 90.32 | 1.83 | .071 | -0.04 | 1.06 |
| FU2 x sex | 0.69 | 0.32 | 82.44 | 2.14 | .035 | 0.05 | 1.34 |
| FU3 x sex | 0.27 | 0.52 | 70.14 | 0.52 | .602 | -0.76 | 1.30 |
| BL x sex* | 0.00 | 0.00 | . | . | . | . | . |
| FU1 x age | -0.07E-3 | 0.03 | 90.29 | 0.00 | .998 | -0.05 | 0.05 |
| FU2 x age | 0.05 | 0.03 | 83.97 | 1.63 | .106 | -0.01 | 0.12 |
| FU3 x age | 0.06 | 0.05 | 70.39 | 1.14 | .259 | -0.04 | 0.16 |
| BL x age* | 0.00 | 0.00 | . | . | . | . | . |
| FU1 x Aβ(1-38) | -0.92 | 0.62 | 92.11 | -1.50 | .138 | -2.15 | 0.30 |
| FU2 x Aβ(1-38) | -2.53 | 0.75 | 90.09 | -3.36 | .001 | -4.02 | -1.03 |
| FU3 x Aβ(1-38) | -3.57 | 1.21 | 74.28 | -2.95 | 0.004 | -5.98 | -1.16 |
| BL x Aβ(1-38)* | 0.00 | 0.00 | . | . | . | . | . |
| FU1 x Ptau181 | 1.54 | 0.54 | 94.38 | 2.85 | .005 | 0.47 | 2.61 |
| FU2 x Ptau181 | 3.29 | 0.66 | 91.75 | 5.00 | <.001 | 1.99 | 4.60 |
| FU3 x Ptau181 | 3.99 | 1.11 | 79.17 | 3.60 | <.001 | 1.79 | 6.20 |
| BL x Ptau181* | 0.00 | 0.00 | . | . | . | . | . |
| **M2** |  |  |  |  |  |  |  |
| Intercept | 0.94 | 3.72 | 106.57 | 0.25 | .802 | -6.44 | 8.31 |
| SCD | -1.19 | 0.20 | 101.59 | -5.83 | <.001 | -1.59 | -0.78 |
| MCI* | 0.00 | 0.00 | . | . | . | . | . |
| FU1 | 1.36 | 3.74 | 90.66 | 0.36 | .718 | -6.08 | 8.79 |
| FU2 | 7.66 | 4.50 | 84.51 | 1.70 | .092 | -1.29 | 16.61 |
| FU3 | 13.88 | 7.19 | 72.34 | 1.93 | .058 | -0.46 | 28.21 |
| BL* | 0.00 | 0.00 | . | . | . | . | . |
| Sex | -0.19 | 0.21 | 104.04 | -0.93 | .357 | -0.60 | 0.22 |
| Age | 0.01 | 0.02 | 102.22 | 0.61 | .546 | -0.02 | 0.05 |
| Edyears | -0.03 | 0.03 | 101.81 | -0.98 | .328 | -0.09 | 0.03 |
| Aβ(1-38) | -0.37 | 0.81 | 106.82 | -0.45 | .653 | -1.98 | 1.24 |
| Aβ(1-40) | 0.28 | 0.90 | 97.84 | 0.32 | .752 | -1.49 | 2.06 |
| Aβ(1-42) | -0.11 | 0.42 | 100.51 | -0.27 | .791 | -0.95 | 0.73 |
| Ptau181 | 0.36 | 0.38 | 105.37 | 0.95 | .346 | -0.39 | 1.10 |
| FU1 x SCD | -0.44 | 0.28 | 91.89 | -1.58 | .117 | -1.00 | 0.11 |
| FU2 x SCD | -0.76 | 0.34 | 84.83 | -2.26 | .027 | -1.43 | -0.09 |
| FU3 x SCD | -1.13 | 0.53 | 69.90 | -2.15 | .035 | -2.18 | -0.08 |
| BL x SCD* | 0.00 | 0.00 | . | . | . | . | . |
| FU1 x MCI* | 0.00 | 0.00 | . | . | . | . | . |
| FU2 x MCI* | 0.00 | 0.00 | . | . | . | . | . |
| FU3 x MCI* | 0.00 | 0.00 | . | . | . | . | . |
| BL x MCI* | 0.00 | 0.00 | . | . | . | . | . |
| FU1 x sex | 0.51 | 0.27 | 91.34 | 1.87 | .064 | -0.03 | 1.04 |
| FU2 x sex | 0.61 | 0.32 | 83.30 | 1.90 | .061 | -0.03 | 1.25 |
| FU3 x sex | 0.17 | 0.51 | 70.08 | 0.33 | .743 | -0.84 | 1.17 |
| BL x sex* | 0.00 | 0.00 | . | . | . | . | . |
| FU1 x Aβ(1-38) | -0.93 | 0.61 | 93.08 | -1.52 | .133 | -2.14 | 0.29 |
| FU2 x Aβ(1-38) | -2.59 | 0.76 | 90.36 | -3.43 | <.001 | -4.10 | -1.09 |
| FU3 x Aβ(1-38) | -3.63 | 1.21 | 74.39 | -3.00 | .004 | -6.04 | -1.22 |
| BL x Aβ(1-38) | 0.00 | 0.00 | . | . | . | . | . |
| FU1 x Ptau181 | 1.54 | 0.54 | 95.54 | 2.87 | .005 | 0.47 | 2.60 |
| FU2 x Ptau181 | 3.32 | 0.66 | 92.32 | 4.99 | <.001 | 2.00 | 4.63 |
| FU3 x Ptau181 | 3.98 | 1.11 | 79.79 | 3.60 | <.001 | 1.78 | 6.18 |
| BL x Ptau181* | 0.00 | 0.00 | . | . | . | . | . |

Abbreviations: MLR = mixed linear regression, M1 = model 1, M2 = model 2, SCD = subjective cognitive decline, MCI = mild cognitive impairment, AD = Alzheimer’s disease dementia, CDR-SoB = clinical dementia rating sum of boxes, Aβ = β-amyloid, pTau181 = tau protein phosphorylated at threonine 181, BL = baseline, FU1/2/3 = follow-up one, two, and three respectively, LR-tests = Likelihood-ratio tests.

Note. MLR M1 and M2 with CDR-SoB as the dependent variable. In both models, the time trend was non-linear (i.e., including time as a categorical variable). LR-tests were conducted to compare the initial linear (i.e., including time as a continuous variable), models with their non-linear counterpart models, and when possible, final linear models were also tested against final non-linear models. Biomarkers were naturally log-transformed.

*reference category

^†^modelled in visit numbers, using dummy coding with baseline visit as reference category.
